# LncRNA-T199678 Mitigates α-Synuclein-Induced Dopaminergic Neuron Injury via miR-101-3p

**DOI:** 10.3389/fnagi.2020.599246

**Published:** 2020-11-24

**Authors:** Lu-Lu Bu, Ying-Yu Xie, Dan-Yu Lin, Ying Chen, Xiu-Na Jing, Yan-Ran Liang, Su-Dan Peng, Kai-Xun Huang, En-Xiang Tao

**Affiliations:** ^1^Department of Neurology, Sun Yat-sen Memorial Hospital, Sun Yat-sen University, Guangzhou, China; ^2^Guangdong Provincial Key Laboratory of Malignant Tumor Epigenetics and Gene Regulation, Sun Yat-sen Memorial Hospital, Sun Yat-sen University, Guangzhou, China; ^3^Department of Neurology, The Eighth Affiliated Hospital, Sun Yat-sen University, Shenzhen, China

**Keywords:** Parkinson's disease, lncRNA-T199678, miRNA-101-3p, dopaminergic neuron injury, α-synuclein

## Abstract

Parkinson's disease (PD) is the second most common neurodegenerative disorder characterized by dopaminergic neuron death and the abnormal accumulation and aggregation of α-synuclein (α-Syn) in the substantia nigra (SN). Although the abnormal accumulation of α-Syn can solely promote and accelerate the progress of PD, the underlying molecular mechanisms remain unknown. Mounting evidence confirms that the abnormal expression of long non-coding RNA (lncRNA) plays an important role in PD. Our previous study found that exogenous α-Syn induced the downregulation of lncRNA-T199678 in SH-SY5Y cells via a gene microarray analysis. This finding suggested that lncRNA-T199678 might have a potential pathological role in the pathogenesis of PD. This study aimed to explore the influence of lncRNA-T199678 on α-Syn-induced dopaminergic neuron injury. Overexpression of lncRNA-T199678 ameliorated the neuron injury induced by α-Syn via regulating oxidative stress, cell cycle, and apoptosis. Studies indicate lncRNAs could regulate posttranscriptional gene expression via regulating the downstream microRNA (miRNA). To discover the downstream molecular target of lncRNA-T199678, the following experiment found out that miR-101-3p was a potential target for lncRNA-T199678. Further study showed that the upregulation of lncRNA-T199678 reduced α-Syn-induced neuronal damage through miR-101-3p in SH-SY5Y cells and lncRNA-T199678 was responsible for the α-Syn-induced intracellular oxidative stress, dysfunction of the cell cycle, and apoptosis. All in all, lncRNA-T199678 mitigated the α-Syn-induced dopaminergic neuron injury via targeting miR-101-3p, which contributed to promote PD. Our results highlighted the role of lncRNA-T199678 in mitigating dopaminergic neuron injury in PD and revealed a new molecular target for PD.

## Introduction

Parkinson disease (PD) is a progressive neurodegenerative disease classified clinically as a movement disorder and pathologically by nigrostriatal dopamine depletion, and the residual dopaminergic neurons contained Lewy bodies with α-synuclein (α-Syn) misfolding and abnormal aggregation (Dorsey et al., 2007; Kalia and Lang, 2015; Poewe et al., 2017). At present, PD cannot be completely cured and existing therapeutic strategies have limited efficacy at the early stages of PD (Charvin et al., 2018). Therefore, it is in urgent need to clarify the pathogenesis of PD and find new therapeutic targets for PD.

Misfolding and abnormal aggregation of α-Syn are the core features of Parkinson's disease (Fayyad et al., 2019). However, the specific mechanism of α-Syn-induced cell damage is still not clear. Exogenous α-Syn induces abnormal aggregation and misfolding of α-Syn in normal cells. Long non-coding RNAs (lncRNAs) play an important role in a variety of physiology and pathology conditions in neurodegenerative diseases. Mounting evidence shows that lncRNAs are demonstrated to be equipped with multiple functions in PD (Chen et al., 2018; Lu et al., 2018; Wang et al., 2018b). To be specific, lncRNAs have been found to regulate α-Syn expression and aggregation (Liu and Lu, 2018; Xu et al., 2018), mitochondrial dysfunction (Yan et al., 2018; Xie et al., 2019), apoptosis (Ding et al., 2019; Lin et al., 2019), neuroinflammation (Fragkouli and Doxakis, 2014), and autophagy (Yan et al., 2018) in PD. In the specimen of the brain, the expression of 87 lncRNAs in the substantia nigra of PD patients was significantly changed (Ni et al., 2017). Among which, lncRNA AL049437 and lncRNA AK021630 were verified, via experimental test, to promote or inhibit the occurrence of PD, respectively. Also, besides brain tissue, abnormal expression of lncRNAs was also found in peripheral blood and cerebrospinal fluid of PD patients. Studies indicated that more than 6,000 lncRNAs were detected in peripheral blood leukocytes of PD patients, of which 13 lncRNAs decreased and among which 4 abnormally expressed lncRNAs were reversed after deep brain stimulation (DBS) (Soreq et al., 2015). Despite many exciting advances in lncRNA biology, the relationship between lncRNAs and PD pathogenesis remains elusive.

In our preliminary study, clear downregulation of lncRNA-T199678 (G046036) was noted in the exogenous α-Syn-induced SH-SY5Y cell model of PD by gene microarray (Lin et al., 2018). Subsequently, we confirmed that the expression level of lncRNA-T199678 was reduced in the α-Syn-induced SH-SY5Y cell model. Yet the function of lncRNA-T199678 in PD pathogenesis has not been rigorously studied. To address this point, in the present study we investigated the profile of lncRNA-T199678 in the α-Syn-induced SH-SY5Y cell model. We found that α-Syn downregulated lncRNA-T199678, which targeted miR-101-3p, thus aggravating neuron injury in PD. Therefore, this study explores the function of lncRNA-T199678 in the etiology and pathogenesis of PD and providing scientific clues for lncRNA-based treatments of this neurodegenerative disorder.

## Materials and Methods

### Cell Culture

SH-SY5Y cells were obtained from the Cell Bank of the Chinese Academy of Medical Science (Shanghai, China). Cells were cultured in Dulbecco's modified Eagle's medium (DMEM; Gibco, Carlsbad, CA, USA) containing 10% fetal bovine serum (FBS; Invitrogen, Carlsbad, CA, USA), 100 U/mL penicillin, and 100 μg/mL streptomycin in a humidified atmosphere with 5% CO_2_ at 37°C.

### Preparation for α-Syn Oligomer

The powder of α-Syn monomer was kindly donated by Professor Yu Shun, Department of Neurology, Xuanwu Hospital, Capital Medical University. The powder was dissolved in 0.01 M PBS to a final concentration of 25 μM (pH = 7.4), filtered and sterilized, and then incubated at 37°C for 72 h with oscillations for 100–150 R/min.

### RNA Extraction and qPCR

The medium was aspirated, and cells were washed once with PBS. RNA was extracted using the Trizol (Takara, Japan) according to kit instructions. The concentration and quality of RNA were detected by NanoDrop ND-1000. cDNA was prepared using the Prime Script™ RT Reagent Kit (Takara, Japan) according to the manufacturer's instructions and diluted 1:10 in double-distilled water before qPCR plate preparation.

qPCR was performed in 96-well plates, using SYBR(R) Premix Ex Taq™. β-Actin was used as a housekeeping gene for SH-SY5Y cells. All data were calculated by the 2^−ΔΔ*Ct*^ method. (Primer sequences were detailed as follows).

**Table:** 

**Primer**	**Sequences**
lncRNA-T199678-R	5′-TCTATTTTGTTGGTTTTCGG-3′
lncRNA-T199678-F	5′-ATGCTTTCGCTCTGGTCT-3′
β-Actin-F	5′-TGTCCACCTTCCAGCAGATGT-3′
β-Actin-R	5′-AGCTCAGTAACAGTCCGCCTAG-3′
hsa-miR-101-3p-F	5′-ACACTCCAGCTGGGTACAGTACTGTGATAAC-3′
hsa-miR-101-3p-R	5′-CTCAACTGGTGTCGTGGA-3′
hsa-miR-101-3p-RT	5′-CTCAACTGGTGTCGTGGAGTCGGCAATTCAGTTGAGTTCA
	GTTA-3′

### *In situ* Hybridization

An appropriate amount of cells was inoculated into confocal dishes and treated in groups. Fixed with 4% paraformaldehyde at room temperature for 10 min, the cells were washed with PBS for 5 min, three times. One microliter precooled permeable solution (Triton-X100, Sigma, USA) was added to each well and stood at 4°C for 5 min. The permeable solution was discarded, and the cells were washed with PBS for 5 min, three times. The following *in situ* hybridization experiments were carried out according to the instructions of fluorescent *in situ* hybridization kit of Guangzhou Ruibo Biotechnology Co., Ltd. 200 μL of the pre-hybrid solution was added to each well and sealed at 37°C for 30 min. At the same time, the hybrid solution was preheated at 37°C. In the dark conditions, a mixed storage solution of 2.5 μl 20 μM lncRNA FISH Probe or control FISH Probe was added to the 100-μl hybrid solution. Then, the pre-hybrid solution was discarded in each well, and a 100-μL probe hybrid solution-containing probe was added overnight at 37°C in the absence of light. Hybrid lotions I, II, and III were preheated to 42°C; the cells were washed with hybrid lotion I for three times, 5 min each time in dark conditions and then washed with hybrid lotion II and hybrid lotion III once, 5 min each time. Next, the cells were washed with PBS, 5 min at room temperature, and 4′,6-Diamidino-2-Phenylindole, Dihydrochloride-stained for 10 min in dark conditions. Cells were washed with PBS for three times, 5 min each time. A fluorescence quenching agent was added under dark conditions (Wuhan Baodu Bio-Engineering Co., Ltd., China), and fluorescence detection was carried out by a confocal microscope (Zeiss LSM, Germany) (maximum excitation light length was 555 nm, maximum emission wavelength was 570 nm).

### RNA Transfection and Interference

LncRNA-T199678-overexpressed plasmid PCDNA3.1-T199678 (PC-T199678) and its negative control (pcDNA3.1), lncRNA-T199678 Smart Silence (siT199678) and its negative control (siNC), and the miR-101-3p mimic and NC mimic were synthesized by Ruibo Biotechnology Co., Ltd. (Guangzhou, China). A Lipofectamine 3000 transfection agent (Invitrogen) was used for transfection according to the specification. The expressions of lncRNA-T199678 and miR-101-3p were detected by qPCR.

### RNA Immunoprecipitation

The RNA immunoprecipitation was performed by using a Magna RIP Kit (Product NO. 17-701, Millipore, USA). In brief, SH-SY5Ycells were cells were lysed by RIP lysis (Shanghai Beyotime Biotechnology Co., Ltd., China), and cell lysates (100 μL) were incubated with RIP buffer containing magnetic beads conjugated with the human anti-Ago2 antibody (1:1000, Abcam 32381) or negative control normal mouse IgG (Millipore) at 4°C overnight. Then immunoprecipitated RNA was extracted. Purified RNA was subjected to further study.

### Detection of Intracellular ROS

Intracellular ROS was detected using an oxidation-sensitive fluorescent probe (DCFH-DA) (Shanghai Beyotime Biotechnology Co., Ltd., China). Cells were incubated with 10 μmol/L DCFH-DA at 37°C for 20 min according to the manufacturer's instructions. The cells were washed three times with serum-free cell culture medium to fully remove DCFH-DA. Fluorescence of DCFH was detected by flow cytometry, and the excitation wavelength of 488 nm and emission wavelength of 525 nm were used to detect the fluorescence intensity before and after stimulation in real time.

### Cell Cycle Analysis

Cell cycles were measured using the cell cycle kit (Product NO. BMS500FI-300) from Thermo Fisher, USA. The cells were digested by pancreatic enzymes. The supernatant was removed by centrifugation at 1,000 rpm for 5 min and then resuspended in PBS and fixed with 2 mL precooled 70% ethanol for 24 h at 4°C. After fixation, the supernatant was removed after centrifugation at 1,000 rpm for 5 min and washed with PBS. The precipitated cells were added with 50 μl RNA enzyme and 450 μl propidium iodide (PI) staining solution for flow cytometry within 4 h.

### Cell Apoptosis Analysis

The apoptosis of the neurons was measured using an Annexin/V-FITC apoptosis detection kit (Product NO. BMS500FI-300, Thermo Fisher, USA) according to the manufacturer's instructions. The neurons were harvested in the EP and spun down for 5 min at 1000 rpm. Cells were washed in ice-cold PBS and resuspended in a 1 × binding buffer. Then 5 μl of Annexin V-FITC was added to the cell suspension and incubated for 15 min at room temperature. Lastly, 10 μl PI buffer was added and analyzed immediately by flow cytometry.

### Statistical Analysis

All *in vitro* experiments were performed in triplicate, and each assay was repeated three times. The mean values from the triplicate measurements were used for statistical analysis. Data were presented as mean ± standard deviation (SD). Comparisons between two groups were performed with Student's *t*-test, and multiple comparisons of more than two groups were performed with one-way ANOVA followed by Tukey's multiple-comparison tests using GraphPad Prism 5. *P* < 0.05 was considered statistically significant.

## Results

### Exogenous α-Syn Induces Stable Downregulation of lncRNA-T199678 Expression in SH-SY5Y Cells

**Figure 1:**
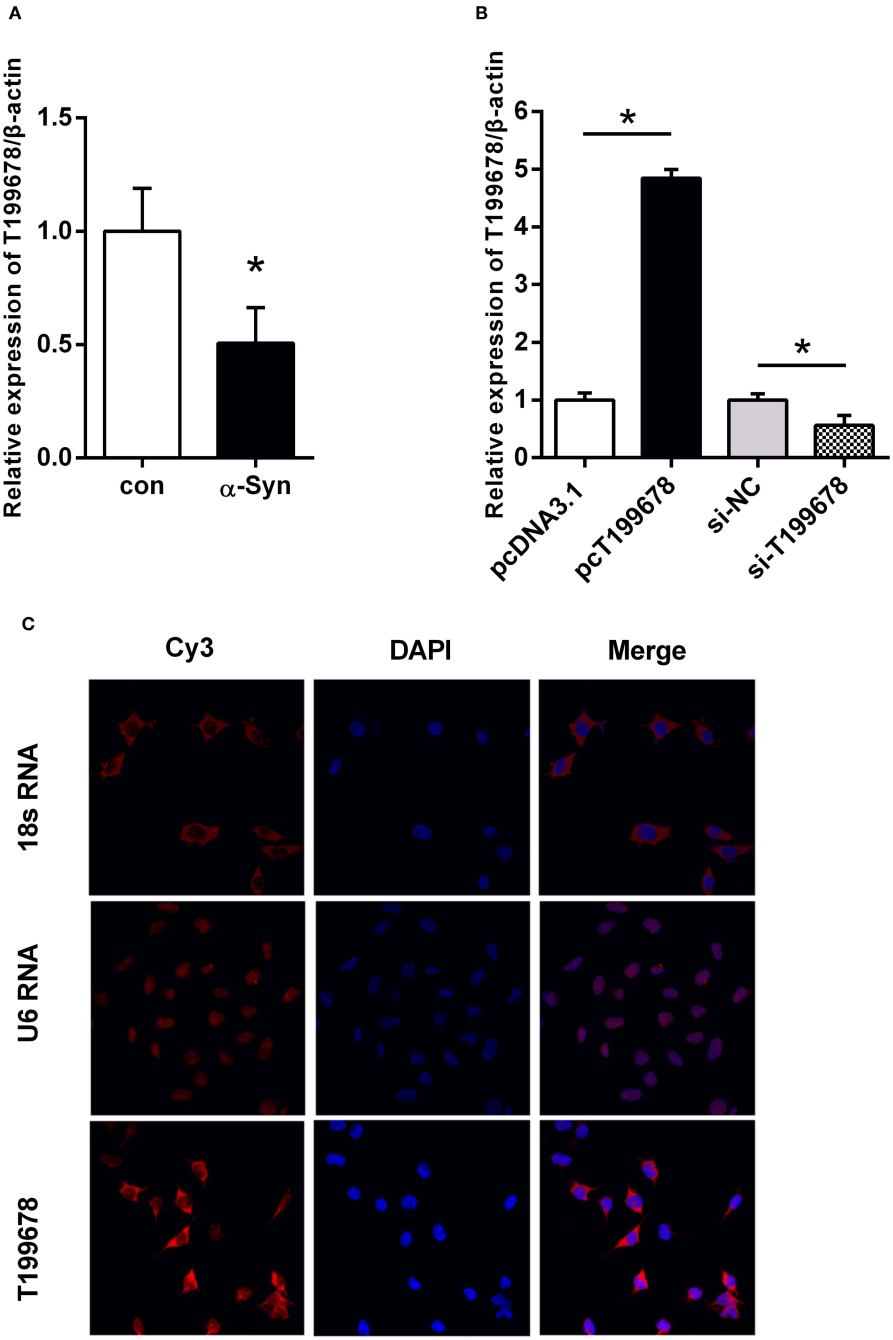
The level and sub-localization of lncRNA-T199678 in α-Syn-challenged SH-SY5Y cells**. (A)** Relative expression of lncRNA-T199678 was markedly decreased in α-Syn-challenged SH-SY5Y cells by qPCR (*p* < 0.05, compared with the control group). **(B)** Relative expression of lncRNA-T199678 in lncRNA-T199678 overexpression and silencing cell lines by qPCR. **(C)** Sub-localization of lncRNA-T199678 in SH-SY5Y cells. Taking U6 located in the nucleus and 18S located in the cytoplasm as the control, most lncRNA-T199678 in the SH-SY5Y cell line were located in the cytoplasm. ^*^P < 0.05.

Our previous research screened a significant downregulated lncRNA-T199678 in the differential expression gene in the pathologically α-Syn-induced group via a gene chip (Lin et al., 2018). To determine whether lncRNA-T199678 was involved in α-Syn-induced dopaminergic neuron damage, the relative expression of lncRNA-T199678 was detected via qPCR. In the α-Syn-challenged SH-SY5Y cell line, the expression of lncRNA-T199678 was markedly decreased when comparing to the control group (Figure 1A, *P* < 0.05). Therefore, to explore the function of lncRNA-T199678, we constructed lncRNA-T199678 overexpression and silencing cell lines as shown in Figure 1B. The lncRNA-T199678 expression was lower in the α-Syn-challenged SH-SY5Y cell line, as examined by RT-qPCR (Figure 1A, *P* < 0.05). These results indicated that lncRNA-T199678 may be a key player in α-Syn-mediated dopaminergic neuron loss.

### Sub-Localization of lncRNA-T199678 in SH-SY5Y Cells

The localization of lncRNA in the cell is closely related to its function. Therefore, we studied the sub-localization of lncRNA-T199678 in the cell. We found that most lncRNA-T199678 in SH-SY5Y cells were located in the cytoplasm via taking U6 located in the nucleus and 18S located in the cytoplasm as the control, and with only a small amount in the nucleus. The results are shown in Figure 1C.

### Overexpressed lncRNA-T199678 Reverses α-Syn-Induced Cell Injury

**Figure 2:**
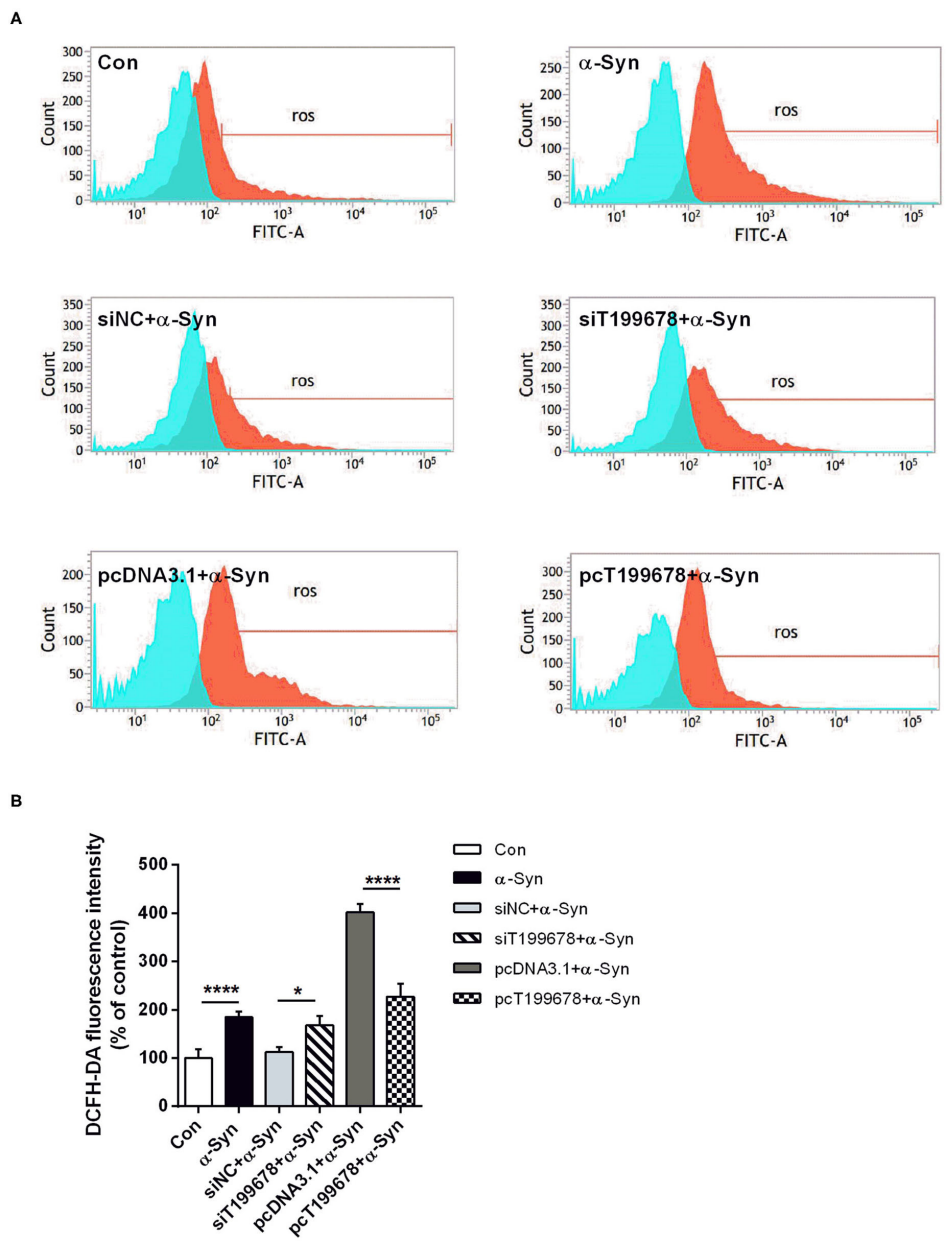
Interference of lncRNA-T199678 ameliorated intracellular oxidative stress induced by α-Syn. SH-SY5Y cells were divided into six groups: control (wild type), α-Syn, siNC+α-Syn, siT199678+α-Syn, pcDNA3.1+α-Syn, and pc T199678+α-Syn. **(A)** Oxidative stress injury (ROS) was detected via DCFH-DA probe and flow cytometry. **(B)** Fluorescence of DCFH-DA was detected by flow cytometry (one-way ANOVA, *F* = 143.1, df = 23, *P* < 0.0001, followed by Tukey's multiple-comparison tests: Control vs. α-Syn, ****P < 0.0001; siNC+α-Syn vs. siT199678+α-Syn, *P < 0.05; pcDNA3.1+α-Syn vs. pc T199678+α-Syn, ^****^P < 0.0001).

**Figure 3:**
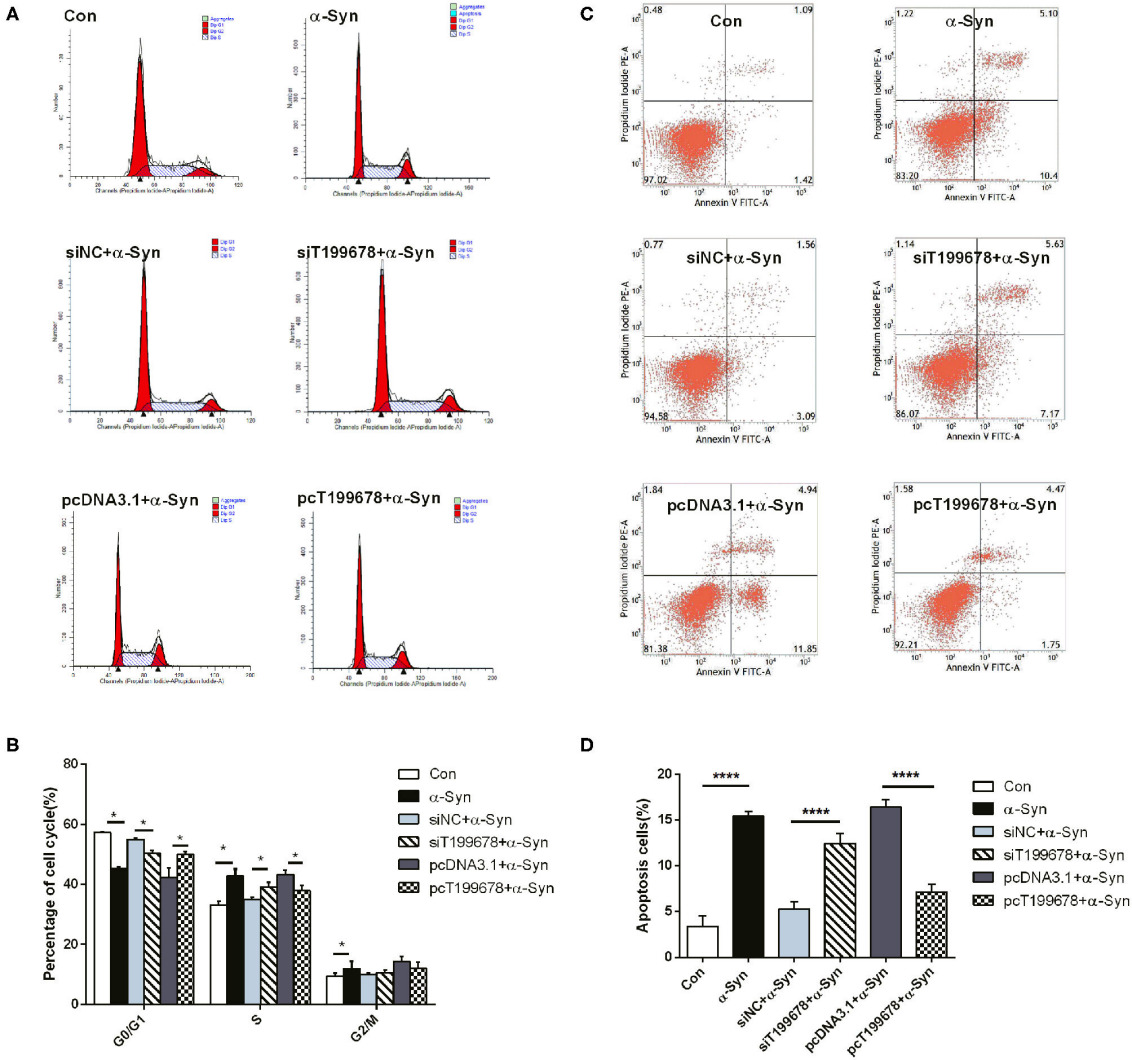
lncRNA-T199678 mitigated α-Syn-induced cell cycle abnormality and apoptosis. SH-SY5Y cells were divided into six groups: control (wild type), α-Syn, siNC+α-Syn, siT199678+α-Syn, pcDNA3.1+α-Syn, and pc T199678+α-Syn. **(A,B)** The proportion of cells in the G0/G1, S, and G2/M phases in cell cycle via flow cytometry (both in the G0/G1 phase and S phase, one-way ANOVA, *P* < 0.01, followed by Tukey's multiple-comparison tests: Control vs. α-Syn, ^*^P < 0.05; siNC+α-Syn vs. si T199678+α-Syn, ^*^P < 0.05; pcDNA3.1+α-Syn vs. pc T199678+α-Syn, ^*^P < 0.05). **(C,D)** Percentage of apoptotic cells was quantified using flow cytometry (one-way ANOVA, *F* = 115.3, df = 17, *P* < 0.0001, followed by Tukey's multiple-comparison tests: control vs. α-Syn, ^****^P < 0.0001; siNC+α-Syn vs. siT199678+α-Syn, ^****^P < 0.0001; pcDNA3.1+α-Syn vs. pc T199678+α-Syn, ^****^P < 0.0001).

To further examine the effect of lncRNA-T199678 on the α-Syn-mediated dopaminergic neuron loss, we constructed lncRNA-T199678 overexpression and silencing cell lines in α-Syn-induced SH-SY5Y cells, followed by detection of the ROS to clear the oxidative stress injury via DCFH-DA probe and flow cytometry. Our results indicated that the level of ROS was significantly increased in the α-Syn-treated group and lncRNA-T199678 silencing group (one-way ANOVA, *F* = 143.1, df = 23, *P* < 0.0001, followed by Tukey's multiple-comparison tests: control vs. α-Syn, ^****^P < 0.0001; siNC+α-Syn vs. siT199678+α-Syn, ^*^P < 0.05; Figures 2A,B), while overexpression of lncRNA-T199678 could reverse intracellular oxidative stress induced by exogenous α-Syn (pcDNA3.1+α-Syn vs. pc T199678+α-Syn, ^****^P < 0.0001; Figures 2A,B). Next, we determined the influence of lncRNA-T199678 on cell cycles, results of which suggested a reverse of cell cycles in an α-Syn-induced cell model with overexpressed lncRNA-T199678 (Figures 3A,B). The data from the cell cycle assay exhibited that the proportion of cells in the G0/G1 phase decreased and that in the S phase increased both in the α-Syn-treated group and in the lncRNA-T199678 silencing group (Figures 3A,B). Then, Annexin V-FITC/PI apoptosis detection kit and flow cytometry were used to measure differences in cell apoptosis. The percentage of apoptotic cells was significantly increased in the α-Syn-treated group and lncRNA-T199678 silencing group (one-way ANOVA, *F* = 115.3, df = 17, *P* < 0.0001, followed by Tukey's multiple-comparison tests: control vs. α-Syn, ^****^P < 0.0001; siNC+α-Syn vs. siT199678+α-Syn, ^****^P < 0.0001; Figures 3C,D), while the apoptosis was proved to be inhibited after overexpression of lncRNA-T199678 along with α-Syn treatment (one-way ANOVA, *F* = 115.3, df = 17, *P* < 0.0001, followed by Tukey's multiple-comparison tests: pcDNA3.1+α-Syn vs. pc T199678+α-Syn, ^****^P < 0.0001; Figures 3C,D). Taken together, we found that lncRNA-T199678 reversed the oxidative stress, cell cycle abnormality, and apoptosis induced by α-Syn. These results indicated that lncRNA-T199678 attenuated the α-Syn-induced dopaminergic neuron damage.

### Interaction Between lncRNA-T199678 and miR-101-3p in SH-SY5Y Cells

**Figure 4:**
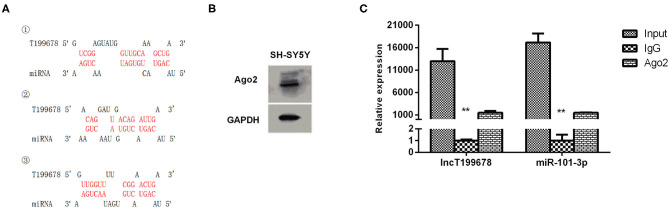
Interaction between lncRNA-T199678 and miR-101-3p in SH-SY5Y cells. **(A)** The potential-binding sites between lncRNA-T199678 and miR-101-3p were shown. **(B)** The expression of Ago2 in SH-SY5Y cells by Western blot. **(C)** Ago2-RNA immunoprecipitation (RIP) followed by qPCR was conducted to determine lncRNA-T199678 endogenously associated with miR-101-3p (^**^P < 0.01).

To investigate the downstream regulatory mechanism of lncRNA-T199678, the interactional binding sites between lncRNA-T199678 and miR-101-3p were predicted via the bioinformatics method (https://bibiserv.cebitec.uni-bielefeld.de/rnahybrid/, RNAhybrid prediction tools, Figure 4A), and the direct binding between them in SH-SY5Y cells was evaluated with RIP (RNA-binding protein immunoprecipitation) assay. Previous studies show that when lncRNA competitively binds miRNA in the form of ceRNA, lncRNA binds to miRNA and RNA-induced silencing complex (RISC), since Ago2 (Argonaute 2) is the core component of RISC, and ceRNA combined with miRNA can also bind to Ago2. We detected the expression of Ago2 in SH-SY5Y cells by Western blot (Figure 4B). Compared with IgG, lncRNA-T199678 and miR-101-3p were abundantly detected in the Ago2 antibody precipitation complex (Figure 4C), which suggested that lncRNA-T199678 had a similar function of ceRNA and could competitively bind to microRNA in the Ago2 complex. Thus, we confirmed the binding of miR-101-3p to lncRNA-T199678, suggesting miR-101-3p as a target of lncRNA-T199678.

**Figure 5:**
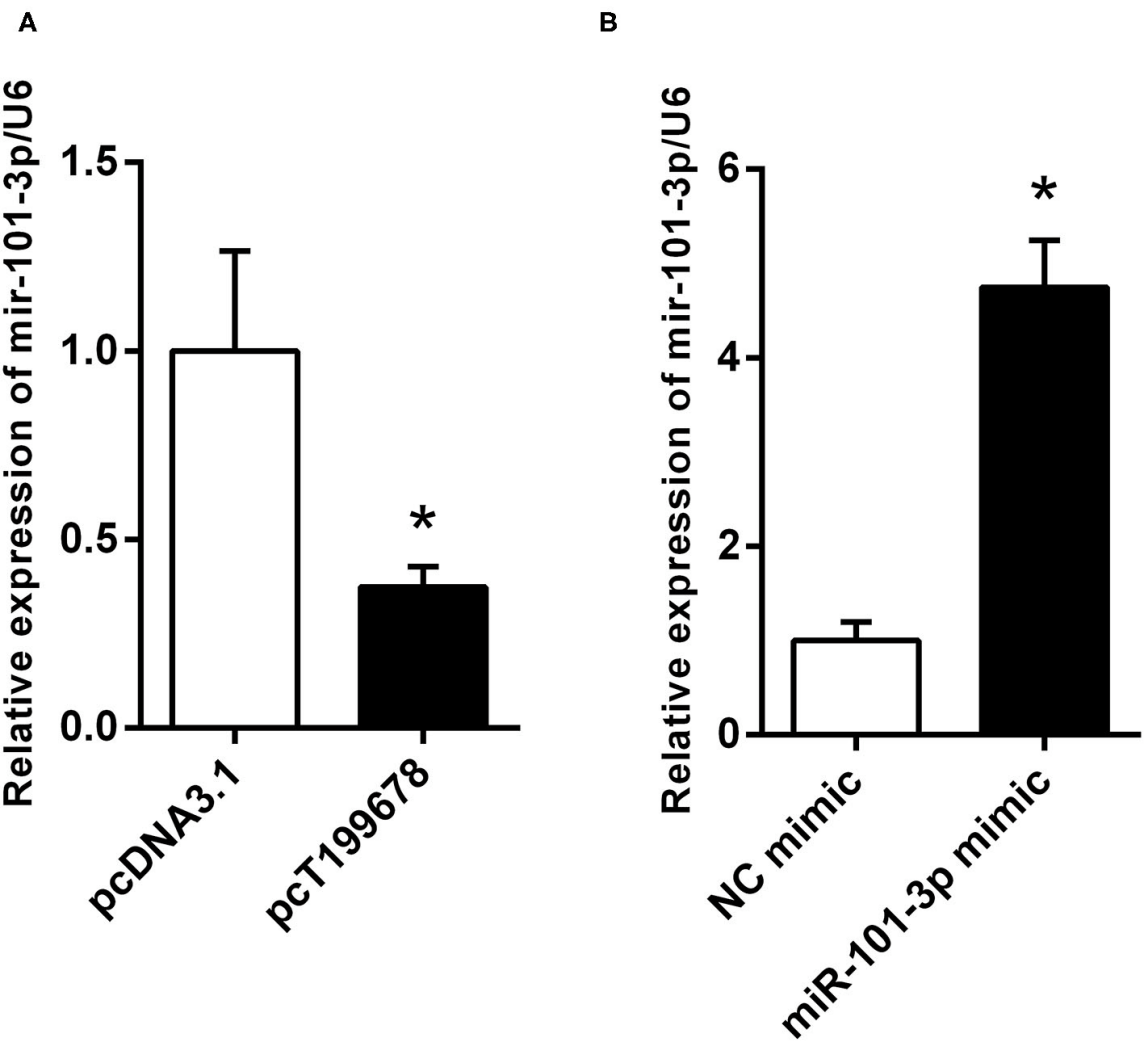
lncRNA-T199678 regulated the expression of miR-101-3p. **(A)** Overexpressed lncRNA-T199678 significantly downregulated the expression of miR-101-3p via qPCR (^*^P < 0.05). **(B)** Construction of overexpressed miR-101-3p in the SH-SY5Y cell line (^*^P < 0.05).

Furthermore, we explored the effects of lncRNA-T199678 on the expression of miR-101-3p by qPCR. Overexpressed lncRNA-T199678 significantly downregulated the expression of miR-101-3p (Figure 5A), and we constructed a miR-101-3p overexpression cell line (miR-101-3p mimic) to study its role in lncRNA-T199678-mediated α-Syn-induced cell damage (Figure 5B).

### Overexpression of lncRNA-T199678 Improved Dopaminergic Neuron Activity That Is Affected by α-Syn Through miR-101-3p

**Figure 6:**
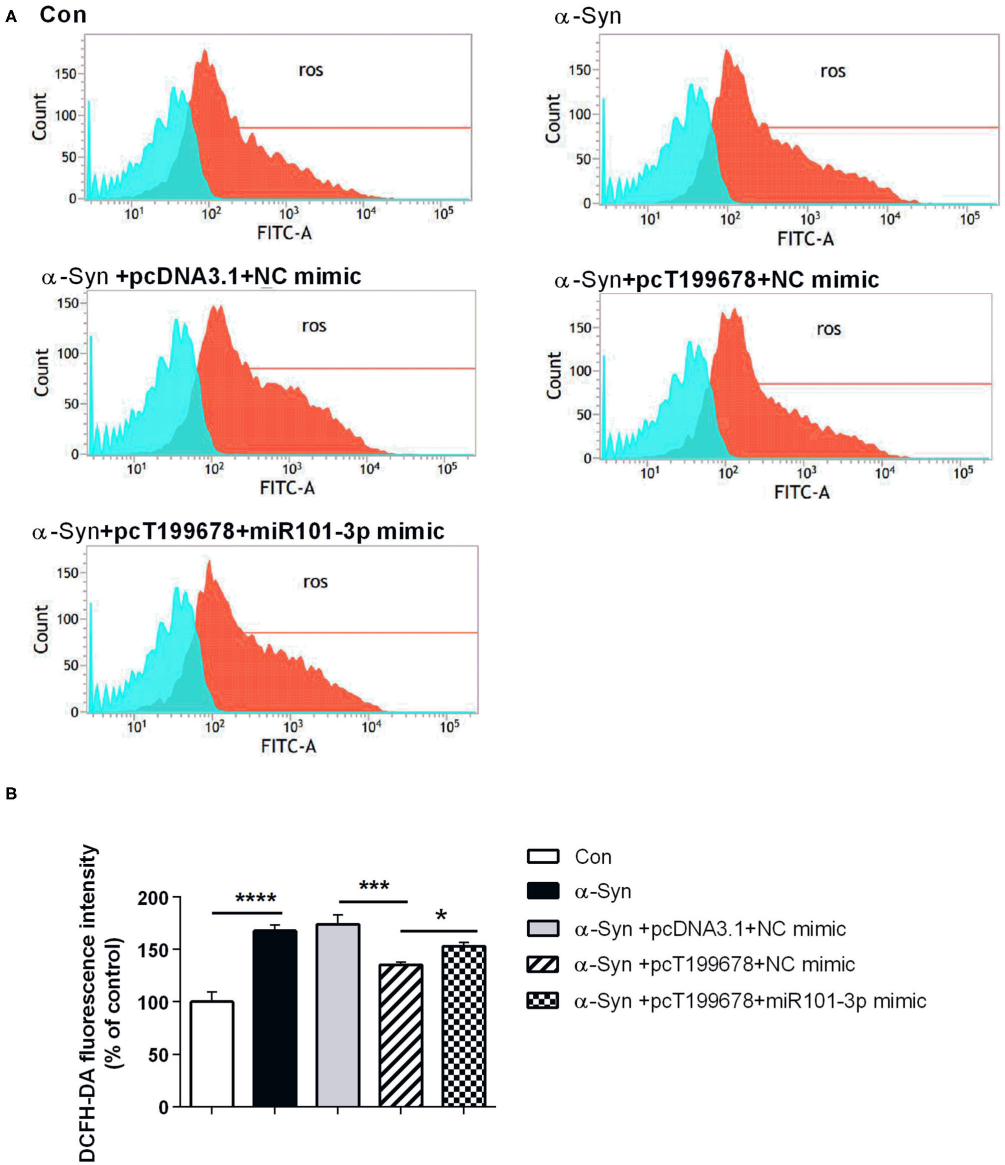
Overexpression of lncRNA-T199678 decreased the ROS production that was affected by α-Syn through miR-101-3p. SH-SY5Y cells were divided into five groups: control (wild type), α-Syn, α-Syn+pcDNA3.1+NC mimic, α-Syn+pcT199678+NC mimic, and α-Syn+pc T199678+ miR-101-3p mimic. **(A)** The oxidative stress (level of ROS) was detected by flow cytometry. **(B)** Fluorescence of DCFH-DA was detected by flow cytometry (one-way ANOVA, *F* = 60.30, df = 14, *P* < 0.0001, followed by Tukey's multiple-comparison tests: control vs. α-Syn, ^****^P < 0.0001; α-Syn+pcDNA3.1+NC mimic vs. α-Syn+pcT199678+NC mimic, ^***^P < 0.001; α-Syn+pcT199678+NC mimic, α-Syn+pc T199678+ miR-101-3p mimic, ^*^P < 0.05).

**Figure 7:**
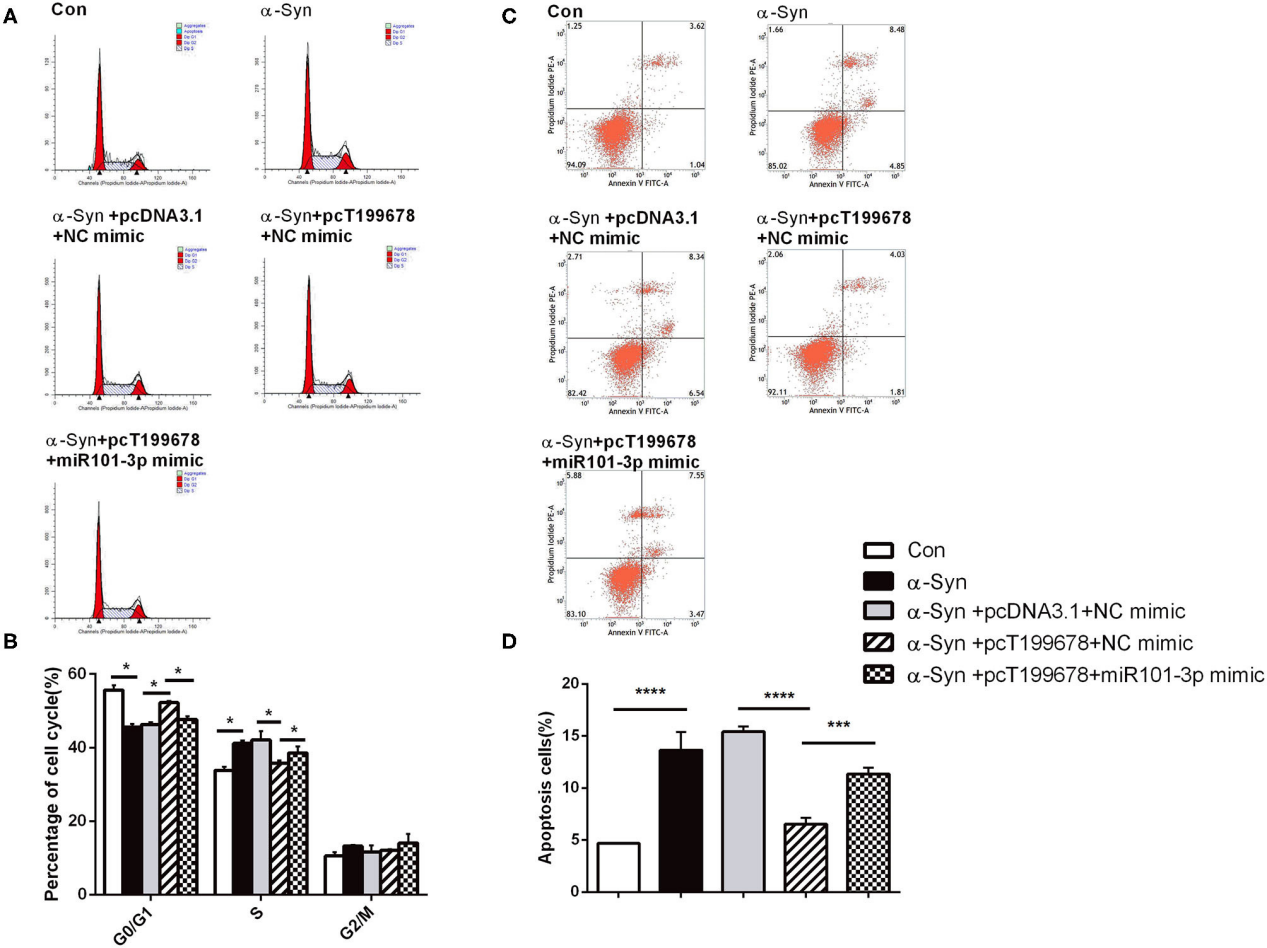
lncRNA-T199678 mitigated α-Syn induced cell cycle abnormality and apoptosis via miR-101-3p. SH-SY5Y cells were divided into five groups: control (wild type), α-Syn, α-Syn+pcDNA3.1+NC mimic, α-Syn+pcT199678+NC mimic, and α-Syn+pc T199678+ miR-101-3p mimic. **(A,B)** The proportion of cells in the G0/G1, S, and G2/M phases in cell cycle via flow cytometry (both in the G0/G1 phase and S phase, one-way ANOVA, *P* < 0.01, followed by Tukey's multiple-comparison tests: control vs. α-Syn, ^*^P < 0.05; α-Syn+pcDNA3.1+NC mimic vs. α-Syn+pcT199678+NC mimic, ^*^P < 0.05; α-Syn+pcT199678+NC mimic, α-Syn+pc T199678+ miR-101-3p mimic, ^*^P < 0.05). **(C,D)** Percentage of apoptotic cells was quantified using flow cytometry (one-way ANOVA, *F* = 76.52, df = 14, *P* < 0.0001, followed by Tukey's multiple-comparison tests: control vs. α-Syn, ^****^P < 0.0001; α-Syn+pcDNA3.1+NC mimic vs. α-Syn+pcT199678+NC mimic, ^****^P < 0.0001; α-Syn+pcT199678+NC mimic, α-Syn+pc T199678+ miR-101-3p mimic,^***^P < 0.001).

Next, we investigated the effect of overexpression of lncRNA-T199678 and miR-101-3p on α-Syn-induced dopaminergic neuron injury. The results showed that the rescue effect of overexpression of lncRNA-T199678 on the damage of dopaminergic neurons induced by α-Syn was halted after co-expression of lncRNA-T199678 and miR-101-3p (Figures 6, 7). Our results indicated that oxidative stress (level of ROS) was significantly enhanced by α-Syn but inhibited after pcT199678 transfection, which was terminally reversed by miR-101-3p mimic (Figures 6A,B). Cell cycle redistribution (mitotic catastrophe) (Figures 7A,B) and neuron apoptosis (Figures 7C,D) were induced by α-Syn but suppressed by overexpression of lncRNA-T199678, but they were both reemerged by a miR-101-3p mimic. It could be concluded that overexpression of lncRNA-T199678 improved α-Syn-damaged neuron activity through miR-101-3p.

## Discussion

Researches on PD have attracted a lot of attention, and abnormal accumulation and aggregation of α-Syn are generally considered as the core hallmark of PD pathogenesis (Spillantini et al., 1997); however, the underlying mechanism is still unclear. Accumulating evidences have illuminated that lncRNAs are abnormally expressed in PD and correlated with its pathology (Wu and Kuo, 2020). In the present study, we identified the downregulation of a novel lncRNA-T199678 in the α-Syn-challenged SH-SY5Y cell lines (Lin et al., 2018). This study is conducted to investigate how lncRNA-T199678 affects the α-Syn-induced neuronal damage by regulating miR-101-3p. Collectively, our study revealed that α-Syn mediated neuron injury by downregulation of lncRNA-T199678. Overexpression of lncRNA-T199678 ameliorated the DA neuron injury induced by α-Syn via regulating oxidative stress, cell cycle, and apoptosis. In the mechanism of action of lncRNA, the regulation network of lncRNA–miRNA–mRNA is the most typical regulation mode of lncRNA. Thus, we further explore the lncRNA–miRNA network and found an interaction between lncRNA-T199678 and miR-101-3p. The following study showed that lncRNA-T199678 mediated α-Syn-induced neuron injury via targeting miR-101-3p, which contributes to improving PD. With this study performed, the pathogenesis of PD was further understood; meanwhile, the potential neuroprotective effect and significance of lncRNA-T199678 in PD were explained, providing novel perspectives into PD progression and treatment.

Accumulating evidences have illuminated that lncRNAs have been participated in disrupting mitochondrial function, regulating α-Syn expression and Lewy body formation, mediating apoptosis, and other mechanisms of PD pathogenesis (Lyu et al., 2019). The lncRNA, NEAT1, regulates the transcription of α-Syn, participates in α-Syn-associated apoptosis (Liu and Lu, 2018), and regulates MPP (+)-induced neuronal injury by targeting miR-124 (Xie et al., 2019). The lncRNA, SNHG14, induces dopaminergic neuron injury by regulating α-Syn via targeting miR-133b (Zhang et al., 2019). LncRNA-p21 prevents the targeting of SCNA mRNA, promotes caspase 3 activation, mediates Bcl family-initiated apoptosis, and regulates neuroinflammation via the upregulation of multiple inflammatory cytokines (Xu et al., 2018; Ding et al., 2019). In the MPTP-treated model, lncRNA MALAT1 associated with α-Syn, leading to the increased stability and expression of α-Syn, exhibiting its crucial role in promoting PD progression (Zhang et al., 2016). In our study, the downregulation of lncRNA-T199678 was identified in the α-Syn-mediated neuron injury, and upregulation of miR-101-3p via binding with lncRNA-T199678 was further involved in the α-Syn-induced dopaminergic neuron injury. It was the first time that the action mechanism of α-Syn was uncovered in PD development by regulating lncRNA-T199678 and the involvement of miR-101-3p in the neuronal injury was also highlighted.

The functions of lncRNAs are closely related to their location. lncRNAs located in the cytoplasm mainly regulate the posttranscription of genes. LncRNAs can directly interact with mRNA to promote or inhibit mRNA translation by stabilizing or promoting the degradation of mRNA. On the other hand, lncRNAs can also act as precursors of miRNAs or bind with miRNAs to prevent transcriptional inhibition of target genes, resulting in increased expression of target genes. LncRNAs located in the nucleus play their roles mainly through epigenetic regulation of chromatin silencing, transcriptional regulation, and selective splicing of pre-miRNAs (Zhang et al., 2014). Our results showed that most lncRNA-T199678 in the SH-SY5Y cell line were located in the cytoplasm (Figure 1C). This demonstrated the direction to explore its function, and the following experiment found miR-101-3p was a potential target for lncRNA-T199678. The etiology of PD has been explained by several hypotheses; loss of dopaminergic neuron is considered one of the most prevalent mechanisms. α-Syn could induce the loss of dopaminergic neurons by different biological processes including oxidative stress, cell cycle regulation, and cell apoptosis. Also, oxidative stress, cell cycle abnormality, and apoptosis are closely related and represent classic, stable, and objective index to reflect the damage of dopaminergic neurons. Pathological α-Syn induces mitochondrial dysfunction, leading to oxidative stress. Meanwhile, ROS can activate ATM, induce DNA damage, and activate the downstream p53 and finally trigger mitochondria-mediated apoptosis. The activation of ATM and DNA damage could drive the cell cycle reinitiation by regulating pRb phosphorylation, and the cell cycle reactivation induces E2F-1 expression and finally mediates cell apoptosis through p53-dependent or non-dependent pathways (Lee et al., 2003; Hoglinger et al., 2007).

Therefore, the alterations of these factors were employed to evaluate the potential function of lncRNA T199678 in the α-Syn-induced cell model. Overexpression of lncRNA-T199678 reversed the α-Syn-induced oxidative stress, cell cycle abnormality, and apoptosis, suggesting that lncRNA-T199678 may play a protective role in the α-Syn-induced dopaminergic neuron damage. Our results suggested that miR-101-3p was identified as a novel target of lncRNA-T199678, hinting that the function of lncRNA-T199678 may be uncovered in more human diseases related to miR-101-3p. LncRNA-T199678 may act as a ceRNA to regulate the expression of miR-101-3p. Our study illustrated the upregulation of miR-101-3p in neuron injury induced by α-Syn, and lncRNA-T199678 promoted α-Syn-induced oxidative stress, cell cycle abnormality, and apoptosis via miR-101-3p. Previous studies showed that miR-101 significantly increased in the striatum of patients with multiple system atrophy. Overexpression of miR-101 led to the accumulation of α-Syn and autophagy defects in oligodendrocytes (Valera et al., 2017). In the PD model, it was found that lncRNA MiRt2 could inhibit TNF-α-induced inflammatory damage by downregulating miR-101 (Han et al., 2019). Apart from its role in PD, miR-101 has been elucidated as a promoter of apoptosis in a variety of cancer cells (Wang et al., 2018a). Besides, miR-101 could enhance the sensitivity of breast cancer cells to oxidative stress by inhibiting the expression of Nrf2, suggesting its role in the process of oxidative stress (Yi et al., 2019). All of the above have shown that miR-101-3p played a key role in the occurrence and development of a variety of diseases including PD.

From the results, it is clear that the downregulation of lncRNA-T199678 promotes dopaminergic dependent oxidative stress injury in PD by upregulating miR-101-3p. Our study offers new clues for the role of the α-Syn/lncRNA-T199678/miR-101-3p axis in PD and emphasizes a new direction for the clinical treatment of PD. Nevertheless, further studies are still required for better elucidation of the specific mechanism of lncRNA-T199678 on PD. Hopefully, lncRNA-based treatments will someday be realized as new medicines to prevent the onset and extend survival in patients with devastating neurodegenerative diseases like PD.

There are several limitations in this study. The specific target genes and signal pathways of miR-101-3p mediated the cell damage need to be further studied. This kind of uncertainty undoubtedly increases the difficulty of miR-101-3p in identifying specific downstream targets in clinical applications. Further studies are needed to identify the downstream genes and pathways mediated by miR-101-3p. Only bioinformatics methods were used to predict the binding sites of lncRNA-T199678 and miR-101-3p, while the specific binding sites still need to be further verified by experimental studies. Moreover, in this study, only cell models were studied; whether α-Syn mediates dopaminergic neuron injury by downregulating the expression of lncRNA-T199678 remains to be further studied in animal models.

## Conclusions

In conclusion, we clarified that a stable low expression of lncRNA-T199678 in α-Syn induced neuron injury, therefore resulting in increased ROS production, abnormal cell cycle, and apoptosis. Furthermore, we found that lncRNA-T199678 mediated α-Syn-induced neuron damage via upregulating miR-101-3p, which contributes to improving PD pathology. Overexpression of lncRNA-T199678 reversed the damage of α-Syn to the neurons via downregulating miR-101-3p. The downregulation of lncRNA-T199678 may be an important mechanism of exogenous α-Syn-mediated dopaminergic neuron injury. Taken together, our study highlighted the initial target of exogenous α-Syn, and the role of lncRNA-T199678 in PD pathogenesis, and emphasized its regulatory relationship with miR-101-3p, providing a new theoretical hypothesis and foundation for the clinical treatment of PD.

## Data Availability Statement

The raw data supporting the conclusions of this article will be made available by the authors, without undue reservation.

## Author Contributions

E-XT and Y-RL designed the experiments and critically revised the manuscript. Y-YX, D-YL, S-DP, and K-XH performed the experiments. L-LB, YC, and X-NJ analyzed and interpreted the data. L-LB and D-YL wrote the manuscript. All authors have read and approved the final manuscript, contributed to the article, and approved the submitted version.

## Conflict of Interest

The authors declare that the research was conducted in the absence of any commercial or financial relationships that could be construed as a potential conflict of interest.
